# Flavored little cigar smoke induces cytotoxicity and apoptosis in airway epithelia

**DOI:** 10.1038/cddiscovery.2017.19

**Published:** 2017-04-24

**Authors:** Arunava Ghosh, Rachel C Nethery, Amy H Herring, Robert Tarran

**Affiliations:** 1 Marsico Lung Institute, University of North Carolina at Chapel Hill, Marsico Hall, 125 Mason Farm Road, Chapel Hill, NC, USA; 2 Department of Biostatistics, Chapel Hill, NC, USA; 3 Department of Cell Biology and Physiology, University of North Carolina, Chapel Hill, NC, USA

## Abstract

Addition of flavors reduces the harsh taste of tobacco, facilitating the initiation and maintenance of addiction among youths. Flavored cigarettes (except menthol) are now banned. However, the legislation on little cigars remains unclear and flavored little cigars are currently available for purchase. Since inhaled tobacco smoke directly exerts toxic effects on the lungs, we tested whether non-flavored and flavored little cigar smoke exposure had the potential for harm in cultured pulmonary epithelia. We cultured Calu-3 lung epithelia on both 96-well plates and at the air–liquid interface and exposed them to smoke from non-flavored Swisher Sweets and flavored (sweet cherry, grape, menthol, peach and strawberry) Swisher Sweets little cigars. Irrespective of flavor, acute little cigar smoke exposure (10×35 ml puffs) significantly increased cell death and decreased the percentage of live cells. Chronic exposure (10×35 ml puffs per day for 4 days) of smoke to Calu-3 cultures significantly increased lactate dehydrogenase release, further indicating toxicity. To determine whether this exposure was associated with increased cell death/apoptosis, a protein array was used. Chronic exposure to smoke from all types of little cigars induced the activation of the two major apoptosis pathways, namely the intrinsic (mitochondrial-mediated) and the extrinsic (death receptor-mediated) pathways. Both flavored and non-flavored little cigar smoke caused similar levels of toxicity and activation of apoptosis, suggesting that flavored and non-flavored little cigars are equally harmful. Hence, the manufacture, advertisement, sale and use of both non-flavored and flavored little cigars should be strictly controlled.

## Introduction

Tobacco smoking and second-hand smoke exposure are major causes of mortality and morbidity worldwide.^1^ According to the National Adult Tobacco Survey (2013–2014), cigars, cigarillos and filtered little cigars constitute the second largest group of consumed combustible tobacco products.^2^ Little cigars are combustible tobacco weighing <3 lbs per 1000 units and are filler wrapped in tobacco leaf or tobacco-containing material. Little cigars have also been perceived as ‘safer’ than cigarettes, although no data exist regarding this.^3,4^ Each day, more than 3800 youths <18 years of age smoke their first cigarette and flavored tobacco products may facilitate smoking.^5^ Flavored tobacco products are especially appealing to younger smokers as they may mask the harsh taste of tobacco, resulting in the initiation, amplification and continued addiction to tobacco products.^6^ Enticing flavors like fruit, menthol, liquor, candy and coffee, along with attractive packaging, help in new tobacco user recruitment.^6^ In the United States of America, with the issuing/implementation by the Food and Drug Administration (FDA) of a finalized rule in May 2016, all tobacco products are subjected to the Federal Food, Drug and Cosmetic Act (the FD&C Act), as amended by the Family Smoking Prevention and Tobacco Control Act (Tobacco Control Act). Hence, little cigar distribution and sales are now controlled, with warning statements being required on packaging. Although only menthol flavoring is allowed in cigarettes, diverse flavors are currently permitted in little cigars. However, the FDA accepts the influence of flavors on initiation and continued use of tobacco products and intends to issue a product standard to eliminate flavors from little cigars (Federal Register/Vol. 81, No. 90/Tuesday, 10 May 2016/Rules and Regulations, pages 28977 and 29024).

Cells typically undergo a complex signaling cascade before undergoing apoptosis/cell death, including release of cytosolic Ca^2+^ and post-translational modification of several proteins, for example, the cleavage of caspase-3, suggesting that proteins in the apoptosis pathway may constitute biomarkers of harm that can be evaluated after tobacco smoke exposure.^7,8^ Furthermore, we have previously demonstrated that cigarette smoke exposure elicits a chronic elevation in intracellular Ca^2+^ levels.^9^ Cytosolic Ca^2+^ serves as a universal second messenger that not only control apoptosis but also influences cell division/growth and gene expression.^10,11^ Furthermore, abnormal Ca^2+^ homeostasis has been linked to several pathologies including lung, prostate, breast, skin and colon cancers.^12^ We and others have previously shown that Calu-3 pulmonary epithelial cells show similar, Ca^2+^-dependent inhibition of the cystic fibrosis transmembrane conductance regulator (CFTR) as seen in humans, suggesting that they are a good model for tobacco exposure.^13,14^ Since this inhibition of CFTR was Ca^2+^-dependent,^9^ we therefore tested the ability of flavored and non-flavored little cigars to induce apoptosis/cell death *in vitro* using the Calu-3 cell line.

Although several studies have investigated the effects of individual flavors on biological systems,^15–20^ the effect of flavored tobacco smoke on human airway epithelia has not been determined. Our present study was designed to evaluate the relative toxicity induced by flavors by exposing cultured airway epithelial cells to regular *versus* flavored little cigar smoke. We also evaluated the autophagic response of epithelial cells to non-flavored and flavored little cigar smoke exposure. Furthermore, changes in different apoptotic proteins after chronic exposure of non-flavored and flavored little cigar smoke were evaluated using an apoptosis protein array system.

## Results

### Acute flavored little cigar smoke exposure causes increased apoptosis and cell death

**Figure 1:**
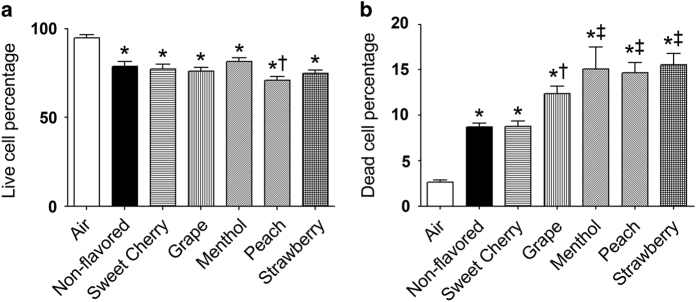
Acute little cigar smoke exposure causes Calu-3 cell death. Calu-3 cells were cultured on 96-well plates and were exposed to 10×35 ml puffs of gas phase smoke from little cigars with or without flavor or air (control). (**a**) Cells were stained with calcein-AM to identify live cells and (**b**) stained with and propidium iodide to identify dead cells; *n*=70 wells per bar. *P⩽0.0001 compared with air. ^†^*P*⩽0.05 and ^‡^*P*⩽0.001 compared with non-flavored little cigars. Bar graphs represent means±S.E.M.

To evaluate the relative effects of non-flavored *versus* flavored little cigars, we exposed Calu-3 cells cultured on 96-well plates to little cigar smoke with air exposure serving as the control. Acute exposure to little cigar smoke (that is, 10 puffs) significantly decreased the percentage of live cells and significantly increased the percentage of dead cells, irrespective of flavoring (Figures 1a and b). Although there was no significant difference between most flavors and the non-flavored brand, peach flavor caused a significantly greater decrease in the percentage of live cells compared with non-flavored smoke. Four out of five flavors tested (grape, menthol, peach and strawberry) also significantly increased cell death compared with the non-flavored little cigar group (Figures 1a and b).

**Figure 2:**
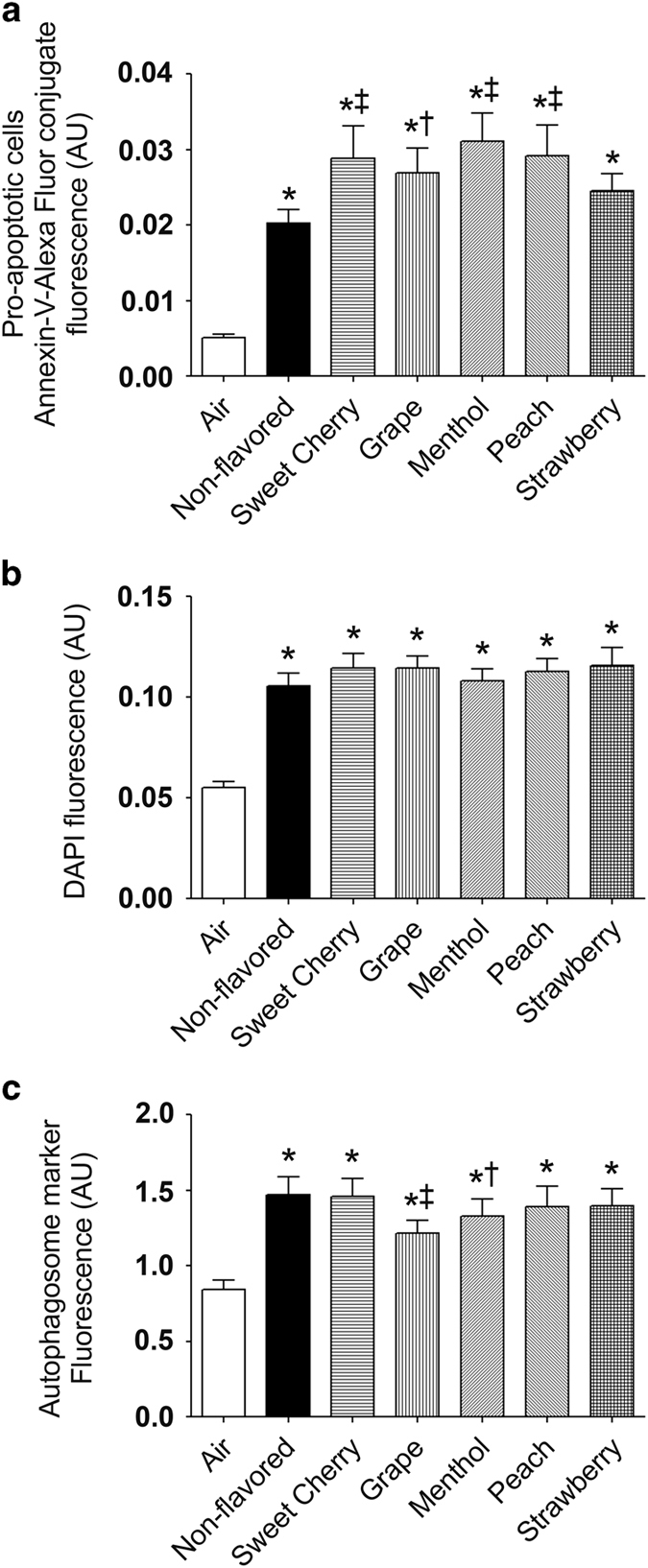
Acute little cigar smoke exposure induces apoptosis and autophagosome formation. Calu-3 cells cultured on 96-well plates were exposed to 10×35 ml puffs of gas phase smoke from little cigars with or without flavor. (**a**) Live Calu-3 cells were stained with Alexa Fluor 647 conjugate to identify proapoptotic cells. (**b**) Cells were stained with DAPI to identify inviable cells. (**c**) Autophagosome formation was identified with a fluorescent autophagosome marker (all *n*=50 wells per bar). *P⩽0.0001 compared with air. ^†^*P*⩽0.05 and ^‡^*P*⩽0.001 compared with non-flavored little cigars. Bar graphs represent means±S.E.M.

Relocation of phosphatidylserine from the inner side of the plasma membrane to the cell surface is an early event of apoptosis that can be detected by the binding of Annexin-V to phosphatidylserine to identify proapoptotic cells.^21^ Additionally, DAPI can be used as a viability exclusion dye in unpermeabilized cells.^22^ To further support our cytotoxicity observations, we stained live unpermeabilized Calu-3 cells cultured on 96-well plates with Annexin-V and DAPI to detect proapoptotic and inviable cells, respectively. The proapoptotic and inviable cells were significantly increased in non-flavored and flavored little cigar smoke-exposed cells compared with the air exposure as indicated by increased Annexin-V and DAPI binding (Figures 2a and b). Four out of five flavors (sweet cherry, grape, menthol and peach) significantly increased Annexin-V fluorescence indicating increased numbers of proapoptotic cells compared with non-flavored little cigar smoke (Figure 2a). No significant difference in DAPI uptake was observed between non-flavored and flavored little cigar smoke groups (Figure 2b).

Acute exposure to both non-flavored and flavored little cigar smoke significantly increased autophagosome formation in Calu-3 cells as detected with an Autophagy Assay Kit (Sigma-Aldrich, MO, USA) (Figure 2c). However, for two out of five flavors (that is, grape and menthol) while the increase was less than for non-flavored tobacco exposure, it was still significantly greater than the air control (Figure 2c). These observations indicate that despite the inclusion of flavors to mask any harsh taste, cytotoxicity remains unaltered.

### Acute flavored little cigar exposure increases cytosolic Ca^2+^ levels

**Figure 3:**
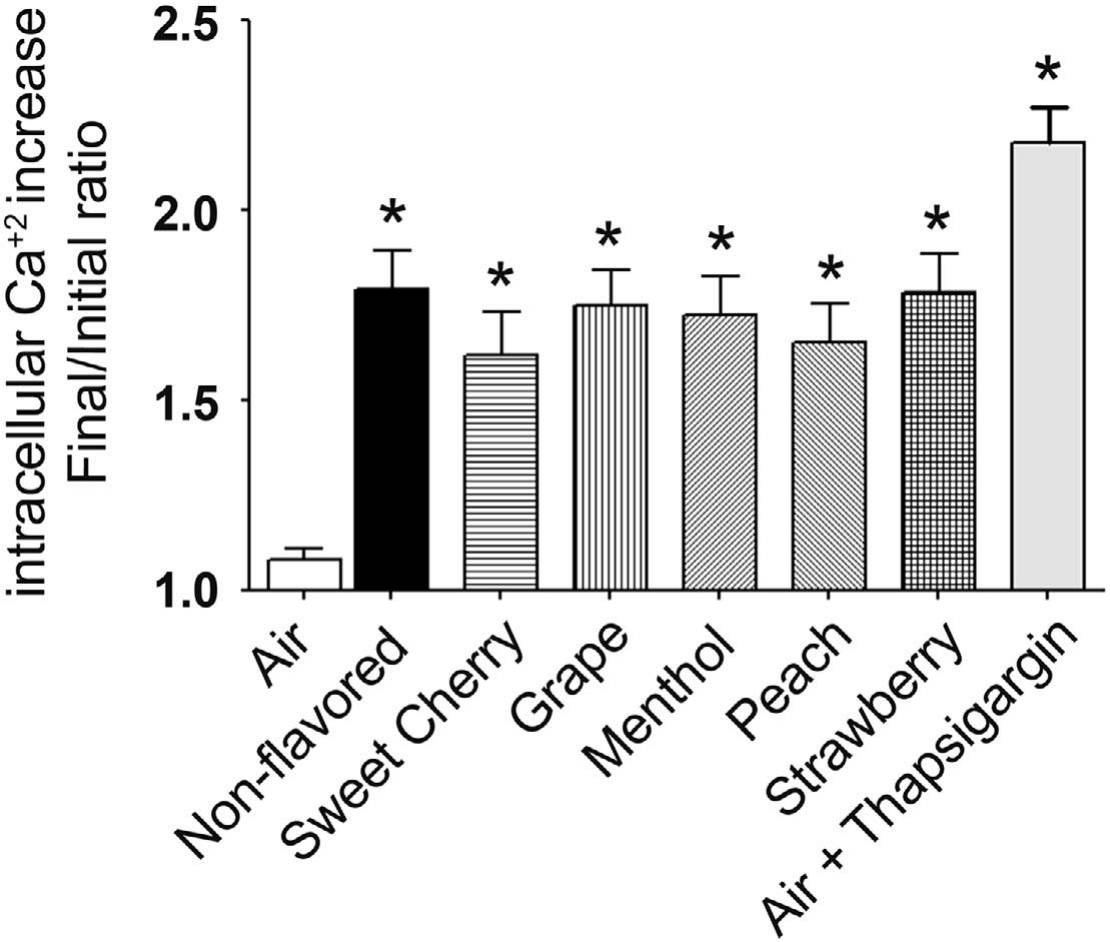
Acute little cigar exposure increases cytosolic calcium levels. Calu-3 cells cultured on 96-well plates were stained with Fluo-4 and fluorescence measurements were recorded both before and after exposure to 10×35 ml puffs of gas phase smoke from little cigars with or without flavor. Intracellular calcium increases were calculated as a ratio of final to initial fluorescence after background subtraction. As a positive control, thapsigargin was added to air-exposed wells (all *n*=42 wells per bar). *P⩽0.0001 compared with air. Bar graph represents means±S.E.M.

Intracellular calcium acts as an important second messenger that regulates cellular signaling including changes in gene expression and the transition to apoptosis.^23^ Cigarette smoke exposure has previously been shown to elicit an increase in intracellular Ca^2+^.^9^ Hence, we measured changes in intracellular Ca^2+^ levels by staining live cells with the calcium-sensitive dye Fluo-4 and monitoring fluorescence before and immediately after 10 puffs of exposure to non-flavored and flavored little cigar smoke. Significant increases in intracellular Ca^2+^ concentrations were observed for all of the smoke exposure groups, irrespective of flavors (Figure 3). However, no significant difference was observed between non-flavored and flavored little cigar smoke exposure (Figure 3). As a positive control, we further added the SERCA pump inhibitor thapsigargin to deplete the endoplasmic reticulum of Ca^2+^ and increase cytosolic Ca^2+^ levels.^24^ These data indicated that little cigar exposure increased cytosolic Ca^2+^ to ~80% of the thapsigargin-induced response (Figure 3).

### Chronic flavored little cigar smoke exposure induces cytotoxicity and increased LC3B-II expression

**Figure 4:**
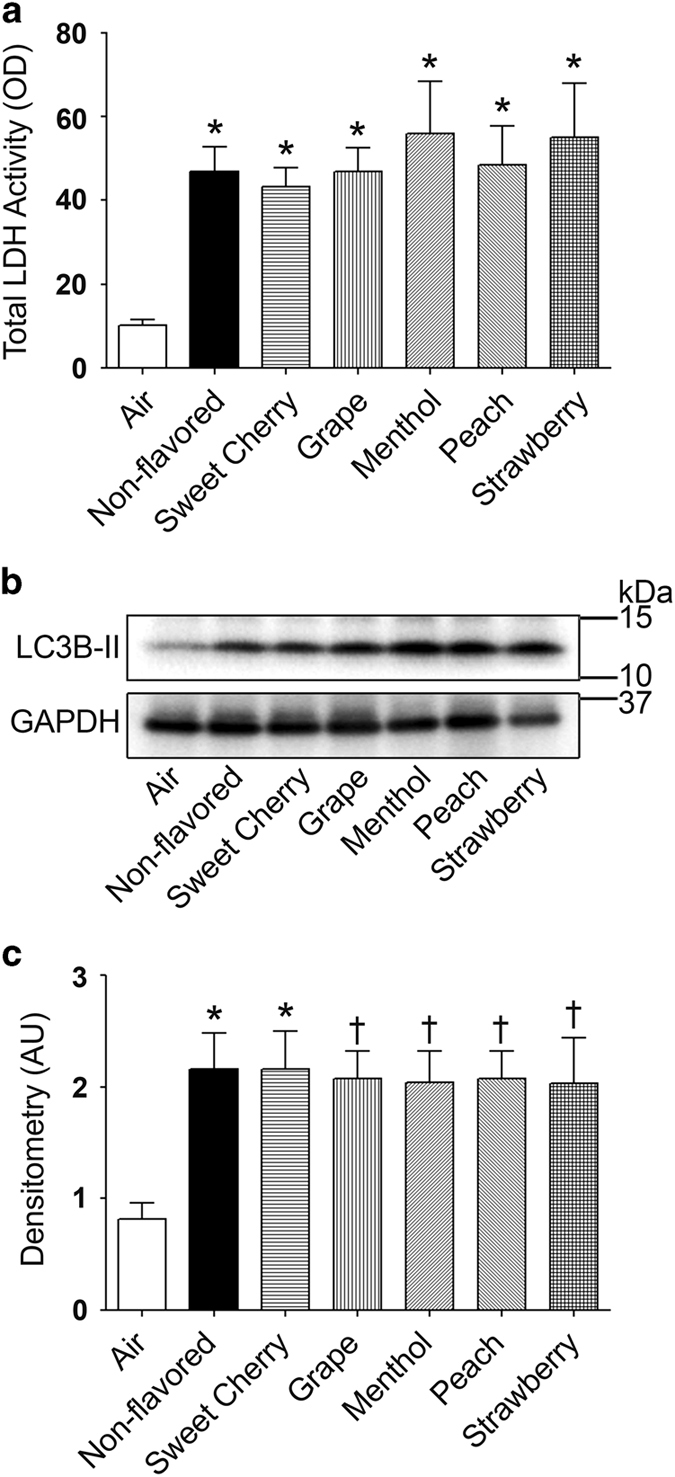
Chronic little cigar smoke exposure induces toxicity and activates autophagy in polarized Calu-3 cultures. Calu-3 cells cultured on transwell-clear inserts were exposed to 10×35 ml puffs per day for 4 days and analyzed for cytotoxicity and autophagic LC3B-II protein expression. (**a**) On the fourth day, LDH release in the basolateral media was increased following chronic tobacco smoke exposure (all *n*=12 cultures per bar). (**b**) Representative western blot of autophagic LC3B-II protein with glyceraldehyde 3-phosphate dehydrogenase (GAPDH) as the loading control. (**c**) Fold change in densitometry (*n*=6 per bar) of LC3B-II protein levels normalized to GAPDH levels after chronic little cigar smoke exposure, with or without flavors. *P⩽0.0001 and ^†^*P*⩽0.05 compared with air. Bar graphs represent means±S.E.M.

For chronic exposure studies, Calu-3 cells were seeded on semipermeable 12 mm transwell inserts and cultured at the air–liquid interface until they were polarized. Cultures were then exposed to 10×35 ml puffs of whole tobacco smoke every day and cytotoxicity was evaluated by measuring lactate dehydrogenase (LDH) release into the basolateral media following the fourth day of exposure. Compared with air-exposed cultures, little cigar smoke exposure, irrespective of flavors, significantly increased LDH release into the basolateral media (Figure 4a). The degree of LDH release was unrelated to flavor and was significantly increased in all the smoke exposure groups (Figure 4a).

Autophagy is associated with programmed cell death and has been implicated in disease pathogenesis.^25^ For example, the lungs of COPD patients display increased autophagy.^26^ Microtubule-associated protein light chain 3 (LC3-II/LC3-I) and autophagy-related proteins (Atg4, Atg5-atg12 and Atg7) are increased during autophagosome formation and can serve as biomarkers of exposure.^26^ Increased levels of LC3B-II protein in the lungs of COPD patients have previously been identified.^27^ Furthermore, *in vitro* tobacco smoke exposure has been shown to alter autophagosome formation and affect the expression of related proteins.^27^ We therefore evaluated the induction of autophagy following chronic exposure to flavored little cigar smoke. We chronically exposed polarized Calu-3 cultures to 10 puffs of whole tobacco smoke per day for four days and measured LC3B-II protein levels. LC3B-II was significantly elevated in all of the chronic smoke-exposed groups irrespective of flavor used (Figures 4b and c). However, no significant difference was observed between flavored and non-flavored little cigar smoke exposures (Figures 4b and c).

### Chronic flavored little cigar smoke exposure alters key apoptotic proteins

**Figure 5:**
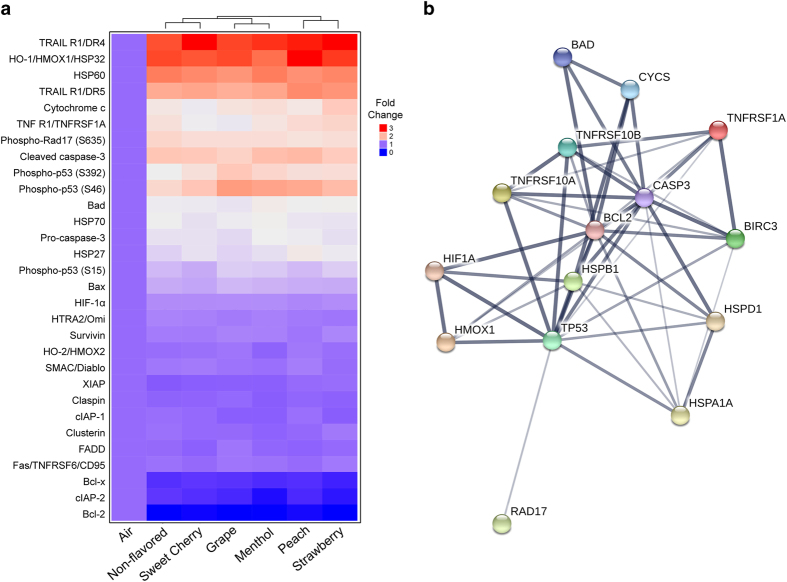
Chronic little cigar exposure induces changes in multiple apoptotic proteins. Polarized Calu-3 cells were chronically exposed to flavored and non-flavored little cigar smoke for 4 days and protein lysate was taken for apoptosis protein array analysis. (**a**) Heat map shows mean changes in expression of apoptotic proteins relative to air. Each value is the mean of four dot blots from two separate runs using lysate pooled from three cultures smoked in two separate experiments. (**b**) String network analysis showing relationship among 15 differentially expressed or phosphorylated apoptosis proteins by chronic little cigar exposure. Each node represents one differentially expressed or phosphorylated apoptotic protein. Line thickness between nodes represents extent of protein interaction.

Apoptotic cell death plays a crucial role in pulmonary disease progression.^28,29^ Apoptosis is driven by both the intrinsic and extrinsic pathways.^7^ While the extrinsic pathway depends on plasma membrane ‘death receptors’, including members of the tumor necrosis factor (TNF) receptor gene superfamily and activation of subsequent signaling cascades, the intrinsic pathway is regulated by mitochondria and relies on the release of specific proteins like cytochrome *c*, high-temperature requirement (HtrA) family serine protease 2 (HTRA2/Omi) and second mitochondria-derived activator of caspases/direct inhibitor of apoptosis binding protein with low pI (Smac/Diablo), which relocate from the mitochondrial intermembrane space into the cytosol to facilitate ‘apoptosome’ formation.^7^ Whether mitochondria release cytochrome *c* is determined by the dimerization of pro- and antiapoptotic members of the Bcl-2 protein family.^7^ The completion of apoptotic cell death is then performed by a group of cysteine proteases known as ‘caspases’, especially caspase-3, which following activation by proteolytic cleavage, degrade diverse groups of proteins to achieve this goal.^7,30^ Other proteins that have critical roles in the activation of apoptosis are p53^31^ and heat-shock proteins (HSPs).^32^ Additionally, ‘inhibitors of apoptosis’ proteins (IAPs) are important regulators of cell death.^33^ As many proteins are involved in apoptosis, we compared the levels of several pro- and antiapoptotic proteins in the whole-cell lysates of chronically smoke-exposed Calu-3 cultures. Out of the 35 proteins tested, we identified the presence of 30 proteins (Figure 5a). Eighteen proteins were altered in chronic smoke-exposed cells. That is, 2 proteins were downregulated and 16 proteins were upregulated after little cigar smoke exposure compared with air (Figure 5a and Table 1). Chronic smoke exposure caused a decrease in the antiapoptotic protein Bcl-2 irrespective of the little cigar flavor (Figure 5a and Table 1). Coupled to this, the proapoptotic mitochondrial protein Bad, which is responsible for mitochondrial membrane pore formation and cytochrome *c* release, was elevated across all groups (Figure 5a and Table 1), indicating that the intrinsic pathway of apoptosis was activated following chronic smoke exposure. Release of cytochrome *c* from mitochondrial membranes into the cytosol leads to the activation of caspases, enabling the execution of apoptosis.^34^ Chronic flavored and non-flavored little cigar smoke exposure increased cytochrome *c* levels compared with air exposure, further demonstrating the activation of apoptosis for all flavors. In contrast, two mitochondrial proteins, namely HTRA2/Omi and Smac/Diablo, remained unaltered.

‘Death receptors’ on the plasma membrane bind to their respective ligands, triggering cell signaling cascades,^7^ leading to caspase activation and activation of the extrinsic pathway of apoptosis. In chronically smoke-exposed cultures, TRAIL R1/DR4, TRAIL R1/DR5 as well as TNF RI/TNFRSF1A receptors were significantly increased (Figure 5a and Table 1) in both flavored and non-flavored smoke exposure groups, indicating that little cigar smoke exposure induced the formation of death receptors irrespective of flavors. However, the protein levels of tumor necrosis factor receptor superfamily member 6 (Fas/TNFRSF6/CD95) receptor and Fas-associated protein with death domain (FADD) remained unaltered across the groups (Figure 5a and Table 1).

Stabilization of p53 by phosphorylation (P-p53) has a pivotal role in the progression towards apoptosis in the event of stresses such as DNA damage.^35^ When whole-cell lysates from chronically smoke-exposed cultures were probed with phosphoserine antibodies against the three phosphorylation sites of p53 (Ser-15, -46 and -392), flavored and non-flavored smoke exposure was found to enhance the phopshorylation of all the three sites, with the effects on Ser-46 being the most profound (Figure 5a and Table 1). Three out of five flavors (grape, menthol and peach) significantly increased S46 phosphorylation compared with non-flavored smoke, with grape flavor showing increased S392 phosphorylation compared with non-flavored smoke (Table 1). Our observations indicate that stress caused by chronic exposure of smoke resulted in stabilization of p53, which subsequently may regulate cell growth and alter transcriptional activation of stress-related genes. Chronic exposure to little cigar smoke regardless of flavor increased phosphorylation of Rad17 at Ser-635 (Figure 5a and Table 1), which is required for genotoxic stress response.^36^

IAP bind to caspases to hinder the progression of cell death.^33^ Three IAP (cIAP-1, XIAP and survivin) remained unaltered across the groups (Figure 5a and Table 1). However, cIAP-2 was found to be downregulated in all groups compared with air (Figure 5a and Table 1), suggesting that the cellular ability to attenuate apoptosis was diminished. For menthol and strawberry flavors, the decrease was significantly greater compared with non-flavored smoke (Table 1).

HSPs are constitutively expressed and act as molecular chaperones to modulate apoptosis in both positive and negative ways.^37^ We analyzed the expression of four HSPs in chronically smoke-exposed cultures, namely HSP27, HSP60, HSP70 and HO-1/HMOX1/HSP32. Previous studies have demonstrated the association of HSPs with tobacco smoke-induced toxicity.^38^ Chronic non-flavored and flavored little cigar smoke exposure caused an increase in the four HSPs tested here, indicating an adaptive cellular response towards the stress induced by tobacco smoke. All the HSPs tested showed increased levels in chronic smoke-exposed cultures irrespective of flavor, with marked increases in HSP60 and HO-1/HMOX1/HSP32 (Figure 5a and Table 1). Furthermore, peach flavor significantly increased HSP27 levels compared with non-flavored smoke (Figure 5a and Table 1).

We further evaluated the levels of procaspase-3 and cleaved caspase-3 in chronic smoke-exposed culture lysates to evaluate the extent of activation of apoptosis following smoke exposure. Both flavored and non-flavored little cigar smoke exposure increased pro- and cleaved caspase-3 levels, signifying apoptosis (Figure 5a and Table 1). Additionally, hypoxia inducible factor-1*α* (HIF-1*α*), a hypoxic transcriptional activator^39^ was elevated in all smoke-exposed cultures (Figure 5a and Table 1). However, claspin, a cell cycle check point protein and clusterin, an antiapoptotic heterodimeric glycoprotein and antiapoptotic enzyme HO-2/HMOX2 levels remained identical across the exposure groups^40–42^ (Figure 5a and Table 1).

Using the list of significantly altered and phosphorylated apoptotic proteins in chronic smoke-exposed cultures, we constructed protein–protein interaction networks using the STRING v.10 algorithm (Figure 5b). The network shows that execution of apoptosis by caspase-3 in chronic smoke-exposed cultures is driven by both the intrinsic and the extrinsic pathways (Figure 5b). The intrinsic pathway was activated following decreased antiapoptotic Bcl-2 protein with increased proapoptotic Bad, leading to cytochrome *c* release. TRAIL death receptors mediated the extrinsic apoptotic pathway in chronic smoked cultures (Figure 5b). Other associated proteins influenced the apoptosis execution indirectly and the interactions between caspase-3 and HSPs were not as strong as those seen with the Bcl-2 family proteins and death receptors (Figure 5b).

## Discussion

In spite of the fact that in the United States of America the Master Settlement Agreement prohibits tobacco companies from targeting youths,^43^ tobacco manufacturers continue to use flavors like fruit, candy, liquor and coffee to recruit new smokers.^44,45^ Indeed, data from the National Youth Tobacco Survey (2011) suggest that more than 40% of middle and high school students in the Unites States of America have used flavored tobacco products.^46^ The effect of flavor compounds on health is a debatable topic. Although individual flavor constituents like anethole, eugenol, pulegone, estragole, piperonal, coumarin and myristicin exert toxic effects on biological systems in high doses,^15–20^ the biological activity of tobacco smoke is unaltered by these ingredients.^47–49^ Furthermore, researchers concluded that flavorants did not affect the relative amounts of ‘Hoffmann analytes’ and did not alter their genotoxicity or cytotoxicity.^50,51^ Similarly, flavored and non-flavored tobacco condensates had similar effects on tumor promotion in SENCAR mouse skin painting bioassays, although here no direct analysis of the mainstream smoke was performed.^52^ Although flavors may not have additional toxic effects *per se*, it has previously been proposed that the addition of flavors to tobacco products is a crucial component in the initiation and sustenance of addiction, suggesting that their investigation is warranted.^44,45,53^

Our current study provides evidence that flavored little cigar smoke causes similar cytotoxicity to pulmonary epithelia as non-flavored little cigar smoke. For example, acute exposure to 10 puffs of little cigar smoke, altered live-dead cell staining, proapoptotic and apoptotic cell staining and autophagosome formation, irrespective of flavor (Figures 1 and 2). We also observed that some flavors were potentially more harmful than others. For example, the menthol, peach and strawberry flavors significantly increased cell death compared with non-flavored little cigars following acute smoke exposure (Figure 1b). Similarly, acute exposure to sweet cherry, menthol and peach flavors significantly increased the formation of proapoptotic cells compared with non-flavored little cigars (Figure 2a). The ability of tobacco smoke exposure to elicit an autophagic response was also evaluated, and we observed that autophagosome formation was enhanced for all flavors (Figure 2c). However chronically exposed air–liquid interface cultures showed uniformly increased LC3B-II protein levels, indicating an increase in autophagy (Figures 4b and c). As all tested flavors induced apoptosis, we then used protein array analysis to evaluate additional apoptotic proteins. We identified similar responses following exposure to smoke from both non-flavored and flavored little cigars, suggesting that all of the little cigars with or without flavor caused activation of both intrinsic and extrinsic pathways of apoptosis (Figures 5a and b).

Cigarette smoke-exposed airway epithelia *in vitro* give similar responses as seen *in vivo*, including inhibition of the CFTR anion channel, increased mucin secretion and induction of apoptosis.^9,14,54,55^ Furthermore, the Calu-3 cell line is widely accepted as a reliable *in vitro* model system of airway epithelia that has been used to predict *in vivo* pharmacological and toxicological effects.^56–58^ Hence, our observations with both adherent and air–liquid interface (ALI) cultures of Calu-3 cells may be used to predict potential *in vivo* implications of flavored and non-flavored little cigar smoke on airway epithelia. Based on our observations, we conclude that little cigars are highly cytotoxic and can induce cell damage and apoptosis. Furthermore, the addition of flavor leaves the cytotoxic effects of the little cigars unaltered, suggesting that they are equally as harmful, despite the mitigating taste effects. In conclusion, considering the appealing effects of these products to new smokers and their potential for toxicity, we propose that flavored little cigars should be regulated in a similar manner as flavored cigarettes.

## Materials and Methods

### Calu-3 cell culture

Calu-3 cells were obtained from the American Type Culture Collection (ATCC, VA, USA) and cultured using Minimum Essential Medium Alpha Medium with 10% fetal bovine serum, penicillin–streptomycin and 1 mM sodium pyruvate. For acute exposure, 40000 cells were seeded per well in 96-well plates (Corning, Corning, NY, USA), incubated overnight at 37 °C/5% CO_2_ and used when the cells were 70–80% confluent. For chronic exposure, 2×10^5^ cells were seeded per 12 mm collagen-coated transwells culture insert (Corning) for 2 days, that is, until they become monolayers, after which time, the apical media was removed to provide an ALI. Cultures were used when they were 7–10 days old.

### Tobacco smoke exposure

All tobacco smoke was generated using an LM1 smoke engine (Borgwaldt, Hamburg, Germany). For acute exposures, cells on 96-well plates were exposed to 10×35 ml puffs of the gas phase of mainstream smoke by placing a polyvinyl alcohol-bound glass fiber filter (Whatman, ISO 3308 compliant), at a frequency of 1 puff per min in a custom-made chamber as described.^14^ For intracellular Ca^2+^ measurements, 1 puff per 30 s was selected, to follow alterations in fluorescence immediately after smoke exposure. For chronic exposures, Calu-3 ALI cultures were exposed to 10×35 ml puffs of whole tobacco smoke per day at a rate of 1 puff per min of a whole little cigar per day for 4 days in a specially designed Perspex smoke chamber before being transferred back into the culture media.^14,59^ During the exposure, cultures were bathed serosally with sterile Ringer’s solution (in mM: 120 NaCl, 5.2 KCl, 1.2 MgCl_2_, 1.2 CaCl_2_·2H_2_O, 12 NaHCO_3_, 24 HEPES, 10 glucose, pH 7.4). Cultures were washed apically with PBS 1 h after the exposure and media were changed daily on all cultures. Control cells were exposed to puffs of filtered air.

### Statistical analysis

All experiments were performed on multiple replicates on a minimum of three separate occasions. For each outcome, we fitted a linear, mixed model with a fixed effect for exposure type and a random effect to account for a possible batch effect within samples run on the same day. These models were used to construct statistical tests to determine whether the effect of air on an outcome was significantly different from the effect of each of the other exposures on that outcome. Similarly, we tested for differences in the effect of non-flavored Swisher Sweets compared with each of the flavored exposures on each outcome. In conducting these tests, a step-down approach was adopted to reduce inflation of the type 1 error. For any given outcome, we first performed an overall test based on its model to determine whether any association with exposure type existed, and follow-up tests for the individual exposure differences of interest were only performed if the overall test was found to be significant at the 0.05 level. *P*-values from the tests of exposure differences of interest were reported for outcomes with significant overall tests.

See Supplementary Methods for details of assays.

## Figures and Tables

**Table 1:** Fold change of 30 apoptotic proteins as determined by microarray in chronic little cigar smoke-exposed polarized Calu-3 cultures, with or without flavor

*Protein*	*Air*	*Non-flavored*	*Sweet Cherry*	*Grape*	*Menthol*	*Peach*	*Strawberry*	P*-value*
Bad	1.00±0.01	1.64±0.11	1.63±0.06	1.60±0.07	1.69±0.07	1.66±0.10	1.67±0.20	**
Bax	1.00±0.01	1.32±0.15	1.29±0.06	1.38±0.07	1.36±0.13	1.33±0.14	1.32±0.17	NS
Bcl-2	1.00±0.03	0.55±0.06	0.59±0.07	0.58±0.08	0.59±0.06	0.60±0.04	0.59±0.08	**
Bcl-x	1.00±0.05	0.72±0.09	0.74±0.07	0.73±0.08	0.70±0.12	0.71±0.12	0.66±0.08	NS
Procaspase-3	1.00±0.01	1.59±0.03	1.59±0.05	1.55±0.07	1.66±0.04	1.64±0.07	1.58±0.08	**
Cleaved caspase-3	1.00±0.09	1.92±0.23	1.90±0.24	1.84±0.25	1.96±0.21	1.99±0.21	1.88±0.19	*
cIAP-1	1.00±0.08	1.01±0.05	0.99±0.06	0.93±0.05	0.93±0.05	1.02±0.09	0.93±0.04	NS
cIAP-2	1.00±0.05	0.75±0.05	0.71±0.02	0.68±0.03	0.61±0.03^a^	0.69±0.05	0.63±0.03^a^	**
Claspin	1.00±0.01	0.96±0.06	0.96±0.02	0.99±0.06	0.93±0.02	0.97±0.09	0.95±0.03	NS
Clusterin	1.00±0.01	1.03±0.07	0.99±0.11	0.99±0.10	0.96±0.11	0.99±0.14	1.07±0.07	NS
Cytochrome *c*	1.00±0.05	1.71±0.08	1.63±0.07	1.73±0.09	1.76±0.16	1.71±0.07	1.88±0.15	**
TRAIL R1/DR4	1.00±0.02	2.55±0.25	2.74±0.28	2.59±0.30	2.66±0.14	2.70±0.28	2.98±0.52	**
TRAIL R1/DR5	1.00±0.00	2.08±0.19	2.11±0.06	2.05±0.08	2.10±0.09	2.26±0.14	2.21±0.11	**
FADD	1.00±0.00	1.00±0.09	0.95±0.02	1.05±0.06	0.98±0.08	0.95±0.08	0.99±0.09	NS
Fas/TNFRSF6/CD95	1.00±0.00	1.03±0.02	0.99±0.04	1.05±0.06	1.03±0.05	1.00±0.03	1.06±0.08	NS
HIF-1*α*	1.00±0.01	1.17±0.04	1.17±0.05	1.17±0.01	1.17±0.03	1.14±0.05	1.17±0.02	**
HO-1/HMOX1/HSP32	1.00±0.06	2.58±0.16	2.53±0.14	2.58±0.13	2.40±0.13	2.73±0.08	2.63±0.25	**
HO-2/HMOX2	1.00±0.02	1.01±0.07	1.02±0.04	1.05±0.05	0.96±0.05	1.07±0.05	1.01±0.03	NS
HSP27	1.00±0.02	1.51±0.09	1.59±0.06	1.52±0.03	1.58±0.09	1.70±0.12^a^	1.63±0.08	**
HSP60	1.00±0.01	2.34±0.11	2.27±0.19	2.20±0.05	2.33±0.09	2.23±0.10	2.29±0.15	**
HSP70	1.00±0.02	1.66±0.12	1.59±0.08	1.64±0.17	1.67±0.12	1.61±0.18	1.60±0.17	**
HTRA2/Omi	1.00±0.02	1.12±0.09	1.11±0.08	1.08±0.09	1.10±0.09	1.07±0.04	1.05±0.02	NS
P-p53 (S15)	1.00±0.01	1.39±0.14	1.32±0.18	1.49±0.17	1.47±0.10	1.39±0.11	1.49±0.08	** * *versus* SC
P-p53 (S46)	1.00±0.02	1.81±0.14	1.91±0.19	2.17±0.21^a^	2.12±0.27^a^	2.08±0.27^a^	1.98±0.20	**
P-p53 (S392)	1.00±0.01	1.65±0.16	1.75±0.17	1.90±0.20^a^	1.81±0.16	1.76±0.12	1.77±0.10	**
P-Rad17 (S635)	1.00±0.02	1.82±0.06	1.77±0.06	1.77±0.11	1.77±0.07	1.74±0.09	1.76±0.06	**
SMAC/Diablo	1.00±0.01	1.06±0.03	1.07±0.03	1.04±0.03	1.02±0.03	1.09±0.05	1.00±0.03	NS
Survivin	1.00±0.02	1.08±0.08	1.08±0.10	1.12±0.06	1.11±0.02	1.05±0.04	1.13±0.05	NS
TNF RI/TNFRSF1A	1.00±0.03	1.75±0.04	1.65±0.14	1.62±0.13	1.73±0.08	1.79±0.07	1.82±0.12	**
XIAP	1.00±0.02	0.91±0.04	0.94±0.09	0.94±0.08	0.94±0.09	1.00±0.13	1.01±0.10	NS

Abbreviations: NS, nonsignificant; SC, Sweet Cherry flavor.

*P*-value column represents comparison of each smoke exposure group compared with air: *⩽0.05; **⩽0.001;

a *P*⩽0.05 for the particular flavor compared with non-flavored smoke.
