# Supplementary material for: Flavored little cigar smoke induces cytotoxicity and apoptosis in airway epithelia
Source: Cell Death Discov. 2017 Apr 24;3:17019–. doi: 10.1038/cddiscovery.2017.19 (PMC5402522; doi:10.1038/cddiscovery.2017.19)
Supplement: Supplementary Methods [file cddiscovery201719-s1.doc]

## Supplementary Methods

### Live-Dead Staining

The distinction between live and dead cell populations was performed by staining with calcein-AM and propidium iodide (PI) respectively. After aspiration of the culture media, cells were washed once with Ringer’s solution and 10 µl Ringer’s solution was added per well. Cells were exposed to smoke and incubated for 5 min before another washing step. 50 µl of staining solution (1 µM calcein-AM, ThermoFisher Scientific and 1.5 µM PI, Sigma; in Ringer’s solution) was added to each well and incubated for 30 min at 37˚C/5% CO_2_. Following another wash step, the fluorescence was recorded using an Infinte M1000 plate reader (Tecan) using excitation and emission wavelengths 495/520 nm for calcein-AM and 530/620 nm for PI respectively. Cultures were then fixed with methanol for 10 min and stained with DAPI (3.6 µM) for 10 min at room temperature and DAPI emission was read at 345/455 nm as excitation and emission wavelengths respectively to normalize calcein-AM/PI to the total number of cells/well. To determine 100% of live cells, wells were incubated with Ringer’s solution for 15 min and to determine the 100% dead cell population, wells were incubated with 70% methanol for 15 min before staining. Data were expressed as percentage of live or dead cells following exposure compared to 100% live or dead cell values.

### Pre-apoptotic and dead cell staining

For the detection of pre-apoptotic and dead cells following the acute exposure protocol, the staining solution used was 1:1000 Annexin V, Alexa Fluor 647 conjugate (Thermo Fisher Scientific) and 1 µg/ml DAPI (Thermo Fisher Scientific). Following the same procedure as mentioned for live-dead staining, the pro-apoptotic and dead cell formation were measured using 650/665 nm for Annexin V and 350/455 nm for DAPI.

### Autophagosome formation detection

Autophagosome formation was detected using an Autophagy Assay Kit (Sigma). After exposing the cells to acute smoke, the staining was followed as per the manufacturer’s protocol and fluorescence emission was read using 360/520 nm as excitation and emission wavelengths respectively.

**Intracellular Ca^2+^ levels**

Increases in intracellular Ca^2+^ concentrations were measured by recording the change in fluorescence of Fluo-4 excited at 490 nm with emission collected at 515 ± 10 nm. Cells seeded on 96 well plates were incubated with 5 µM Fluo-4 (Thermo Fisher Scientific) with PowerLoad solution (Thermo Fisher Scientific) and 2.5 mM Probenecid (Sigma) in media (Minimum Essential Medium (MEM) Alpha Medium with 10% fetal bovine serum, penicillin-streptomycin and 1 mM sodium pyruvate). Following incubation for 30 min at 37˚C/5% CO_2_, baseline fluorescence was recorded. Cells were exposed to 10 puffs of little cigar gas phase smoke (1 puff per 30 second). Fluorescence measurements were then taken immediately after the last puff. The sarco/endoplasmic reticulum Ca^2+^-ATPase (SERCA) pump inhibitor Thapsigargin (Sigma) was added as a positive control to inhibit SERCA mediated increase of intracellular calcium concentration increase to the air exposed wells up to a final concentration of 5 µM and fluorescence reading was recorded again after 5 minutes. Data is represented as ratio of final vs initial Fluo-4 fluorescence.

### Western blotting

For western blotting of light chain 3B-II (LC3B-II) protein cells were lysed with lysis buffer (50 mM Tris pH 7.4, 150 mM NaCl, 1% NP-40, 1 mM EGTA with protease inhibitor cocktail) on ice for 15 min and lysate was centrifuged at 10,000 x g for 5 min at 4˚C. Protein content was measured using the BCA assay and 15 µg of protein was resolved on a 4-15% SDS PAGE gel (Mini-PROTEAN® TGX™ Precast Gels) and transferred to PVDF membrane (Immun-Blot®PVDF Membranes for Protein Blotting). Blots were then blocked with 5% BSA in TBST and primary antibody (Cell Signaling LC3B-II rabbit polyclonal antibody) incubation was performed overnight at 4˚C. Following secondary antibody incubation (Jackson ImmunoResearch donkey anti-Rabbit IgG HRP), blots were developed using Clarity ECL Western Blot Substrate (Bio-Rad) in a ChemiDoc MP imaging system (Bio-Rad). Membranes were stripped using 200 mM Glycine, 0.1% SDS, 1% Tween-20, pH 2.2 and reprobed for GAPDH (Cell Signaling GAPDH rabbit monoclonal antibody) as a loading control. Densitometry of the blots was performed using NIH ImageJ software.

### LDH release

One hour after the 4^th^ exposure (i.e. on day 4), the media was collected and used for detection of LDH release from the cultures using Cytotoxicity Detection Kit (LDH; Roche) following manufacturer’s instructions to detect cytotoxic effects by chronic smoke exposure.

### Apoptosis Array

Proteins involved in apoptosis were analyzed using the Proteome Profiler Human Apoptosis Array following manufacturer’s protocol (R&D Systems). Briefly, following the 4th day of exposure the cultures were washed and lysed using the reagents provided with protease inhibitors. Cultures were rocked slowly at 4˚C for 30 min. Lysate was collected and centrifuged at 14,000 x g for 5 min. Supernatant was frozen immediately at -80˚C and stored until ready. After the lysate was thawed on ice, the protein content was measured using BCA assay and 400 µg of protein was used for array. The membrane was developed in the ChemiDoc MP imaging system (Bio-Rad). Images were analyzed using NIH ImageJ software.

### Protein-protein interaction network

Protein-protein interaction networks were constructed using the STRING v10 algorithm (http://string-db.org), using the names of the proteins altered or phosphorylated in the chronic little cigar smoke exposed cultures as identified by apoptosis array.
